# Pharmacogenomics-guided therapy for essential hypertension: a multi-center randomized controlled open-label trial

**DOI:** 10.3389/fcvm.2026.1842597

**Published:** 2026-09-18

**Authors:** Sha Wu, Wenwu Liu, Long Chen, Yonglong Xu, Jing Jin, Fang Chen, Jun Li, Wuhui Hu, Jie Wu, Yongjun Cai, Zi Huang, Xiaoli Chai, Xiangjiang Shi, Yanlin Luo, Gaofeng Zeng, Haiyan Zeng, Zhi Chen, Fugang Guo, Lei Wang, Mingxin Wu, Zhihao Qu, Yu Wang, Daoquan Peng

**Affiliations:** 1 Department of Cardiovascular Medicine, Research Institute of Blood Lipids and Atherosclerosis, The Second Xiangya Hospital of Central South University, Changsha, China; 2 Department of Cardiovascular Medicine, Linxiang People's Hospital, Linxiang, China; 3 Department of Neurology, Yiyang People's Hospital, Yiyang, China; 4 Department of Cardiovascular Medicine, The Fourth Hospital of Changsha, Changsha, China; 5 Department of Cardiology, Yiyang Central Hospital, Yiyang, China; 6 Department of Neurology, Yiyang Central Hospital, Yiyang, China; 7 Department of Neurology, Taojiang County People's Hospital, Taojiang, China; 8 Department of Cardiovascular Medicine, The First Affiliated Hospital of University of South China, Hengyang, China; 9 Department of Cardiovascular Medicine, Yueyang Traditional Chinese Medicine Hospital, Yueyang, China; 10 Department of Cardiovascular Medicine, Miluo People's Hospital, Miluo, China; 11 Department of Cardiovascular Medicine, The Second People's Hospital of Hunan Province/Brain Hospital of Hunan Province, Changsha, China; 12 Department of Cardiovascular Medicine, The Central Hospital of Shaoyang, Shaoyang, China; 13 Department of Cardiovascular Medicine, The Second Affiliated Hospital of University of South China, Hengyang, China; 14 Department of Hypertension, Changsha Xingsha Hospital, Changsha, Hunan, China; 15 Department of Cardiovascular Medicine, Xiangtan Central Hospital, Xiangtan, China; 16 Hunan Agen Medicine Laboratory Technology Co., LTD, Changsha, China

**Keywords:** antihypertensive therapy, essential hypertension, personalized medicine, pharmacogenomics, randomized controlled trial

## Abstract

**Background:**

The advent of pharmacogenomics-guided antihypertensive therapy represents a paradigm shift in hypertension management. This study aimed to investigate the prevalence of relevant genetic variants among hypertensive patients in Hunan Province, China, and evaluate the efficacy of genotype-directed vs. clinically guided therapy.

**Methods:**

A total of 2,702 patients with essential hypertension from 14 hospitals were enrolled and evenly randomized to either a control group receiving standard care or an experimental group receiving pharmacogenomics-tailored medication. Genetic variants in seven gene loci (*ADRB1*, *CYP2D6*, *CYP3A5*, *CACNA1C*, *CYP2C9*, *ACE*, *ADD1*) associated with five major drug classes were analyzed. Blood pressure and biochemical parameters were assessed at baseline and 4 weeks post-intervention.

**Results:**

The frequencies of the risk allele frequencies for *ACE*, *CYP2C9*, *ADRB1*, *CYP2D6*, *CYP3A5*, *CACNA1C*, and *ADD1* were 34.3%, 4.8%, 74.8%, 57.3%, 69.8%, 11.9%, and 46.3%, respectively. After 4 weeks, blood pressure significantly decreased in both groups (all *P* < 0.001), with a more pronounced reduction observed in the experimental group (from 155.45/92.12 mmHg to 129.03/76.96 mmHg) compared to the control group (from 156.52/91.75 mmHg to 143.74/82.73 mmHg). The genotype-guided group also achieved a significantly higher blood pressure control rate.

**Conclusion:**

These findings highlight the critical role of genetic profiling in antihypertensive drug selection. Personalized treatment based on pharmacogenomics offers a more effective strategy for hypertension management. In the studied Hunan population, angiotensin II receptor blockers and beta-blockers emerged as particularly efficacious options under genotype guidance.

## Introduction

Hypertension, commonly referred to as high blood pressure (HBP), is diagnosed when an individual's office systolic blood pressure (BP) averages ≥140 mmHg and diastolic BP ≥ 90 mmHg, or when they are on antihypertensive drug treatment. It is a leading preventable risk factor for cardiovascular diseases (CVD) and all-cause mortality globally (1). A decade ago, in 2010, hypertension affected 31.1% of the global adult population, translating to 1.39 billion individuals (2). While its prevalence has slightly decreased in high-income countries (HICs) over the past twenty years, a significant increase has been observed in low and middle-income countries (LMICs) (2). Despite these trends, awareness, treatment, and control of hypertension remain alarmingly low, especially in LMICs, where only a third of those affected are aware of their condition, and a mere 8% have their blood pressure under control. This escalating health burden not only drives up mortality rates but also exacerbates social issues, widens health disparities, and imposes a heavy burden on national healthcare systems (3). There is an urgent need to enhance the management of hypertension in these regions.

The primary objective of hypertension treatment is to manage blood pressure and mitigate the progression of CVD, thereby reducing the risk of related morbidity and mortality. The current approach to hypertension treatment is largely a one-size-fits-all strategy, with antihypertensive medications prescribed following clinical guidelines (4). However, given the considerable variability in drug responses due to genetic, environmental, and lifestyle factors, achieving optimal blood pressure control can be challenging (5). This variability may result in inadequate blood pressure control, increased side effects, and a heightened risk of cardiovascular events (6, 7). Thus, there is a critical need for a more targeted approach to hypertension management. Personalized medicine, with a particular focus on pharmacogenomics, presents a promising path for refining antihypertensive therapy. Pharmacogenomics involves the study of DNA variations and gene expression regulation to predict an individual's response to drugs, enabling healthcare providers to select the most effective antihypertensive agents for each patient. This can improve treatment outcomes and minimize complications, potentially reducing side effects and healthcare costs (8).

Pharmacogenomics is gaining traction as a pivotal tool for personalized medication therapy, especially for drugs like neuropsychiatric medications, antitumor agents, and antiplatelet drugs, where genetic testing is deemed essential before prescribing (9). A growing body of research underscores the significance of pharmacogenomics in hypertension management. Studies indicate that integrating pharmacogenomic data into treatment decisions can enhance blood pressure control, lower the incidence of treatment-resistant hypertension, and diminish drug-related adverse effects (10). However, research on the efficacy and safety of pharmacogenomics-based personalized treatment for patients with essential hypertension is still scarce.

Several pharmacogenomic studies have identified key genetic variants that influence responses to antihypertensive drugs. For instance, the ACE insertion/deletion (I/D) polymorphism affects ACE inhibitor efficacy, with the D allele associated with higher ACE activity and better blood pressure control in Asian populations (11–15). The *CYP2C9* polymorphism (particularly the *3 allele) impairs losartan activation, reducing its antihypertensive effect (16, 17). Variants in ADRB1 (encoding β1-adrenergic receptor) and CYP2D6 (involved in metoprolol metabolism) significantly modulate beta-blocker response (18–20). For calcium channel blockers, CYP3A5 and CACNA1C variants have been reported to affect amlodipine efficacy (21–23). The ADD1 (Gly460Trp) polymorphism influences hydrochlorothiazide response by altering renal sodium handling (24, 25). Despite these established associations, large-scale randomized controlled trials evaluating the clinical utility of pharmacogenomics-guided antihypertensive therapy remain limited, particularly in Chinese populations.

This study aims to investigate the efficacy and safety of pharmacogenomics-guided personalized treatment for essential hypertension by adjusting medication use.

## Methods

### Participants

A total of 2,702 hypertensive patients were enrolled in this study from 14 hospitals across Hunan Province between 2021 and 2024. The demographic composition of the sample was 1,536 men and 1,166 women, with a mean age of 60.72 ± 12.09 years. To be included in the study, participants had to meet the following criteria: (I) be over 18 years of age, (II) have a diagnosis of hypertension, and (III) exhibit systolic blood pressure (SBP) ≥ 140 mmHg and/or diastolic blood pressure (DBP) ≥ 90 mmHg on at least three separate occasions, as per the “Revised Guidelines for the Prevention and Treatment of Hypertension in China 2010”. Participants were excluded if they had any of the following conditions: (I) secondary hypertension, (II) concurrent liver or kidney dysfunction, (III) severe heart failure classified as New York Heart Association (NYHA) class III or IV, (IV) poorly controlled diabetes or a history of stroke within the last three months, (V) severe trauma or having undergone major surgery recently. Participants with incomplete essential data (including missing key variables such as baseline blood pressure, genetic sample, or biochemical parameters) were excluded prior to randomization. Participants were randomly assigned to one of two groups in a 1:1 ratio (Figure 1). The experimental group, comprising 1,351 individuals, received antihypertensive treatment tailored according to their genetic profiles, while the control group, also consisting of 1,351 individuals, received treatment based on standard evidence-based medicine (Figure 2). The study protocol was designed in compliance with the Declaration of Helsinki and was approved by the Clinical Research Ethics Committee of the Second Xiangya Hospital of Central South University (Approval No. LYG2021041). Informed consent was obtained from all subjects prior to their participation.

**Figure 1:**
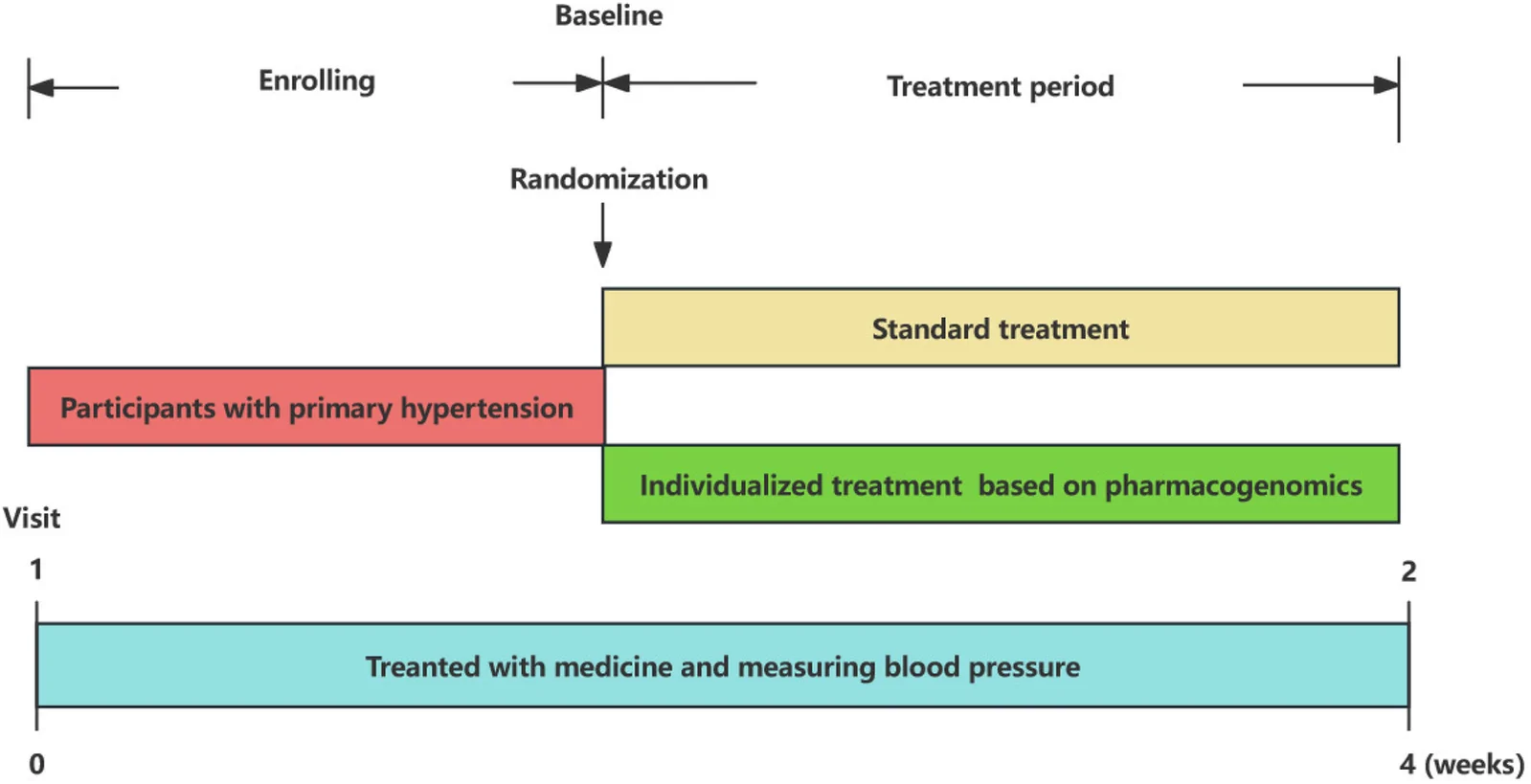
Study design and treatment protocol. Participants with essential hypertension were enrolled and randomly assigned (1:1) to either the control group (standard care) or the experimental group (pharmacogenomics-guided therapy). The treatment period lasted 4 weeks, with blood pressure measurements and biochemical assessments at baseline (week 0) and at the end of treatment (week 4).

**Figure 2:**
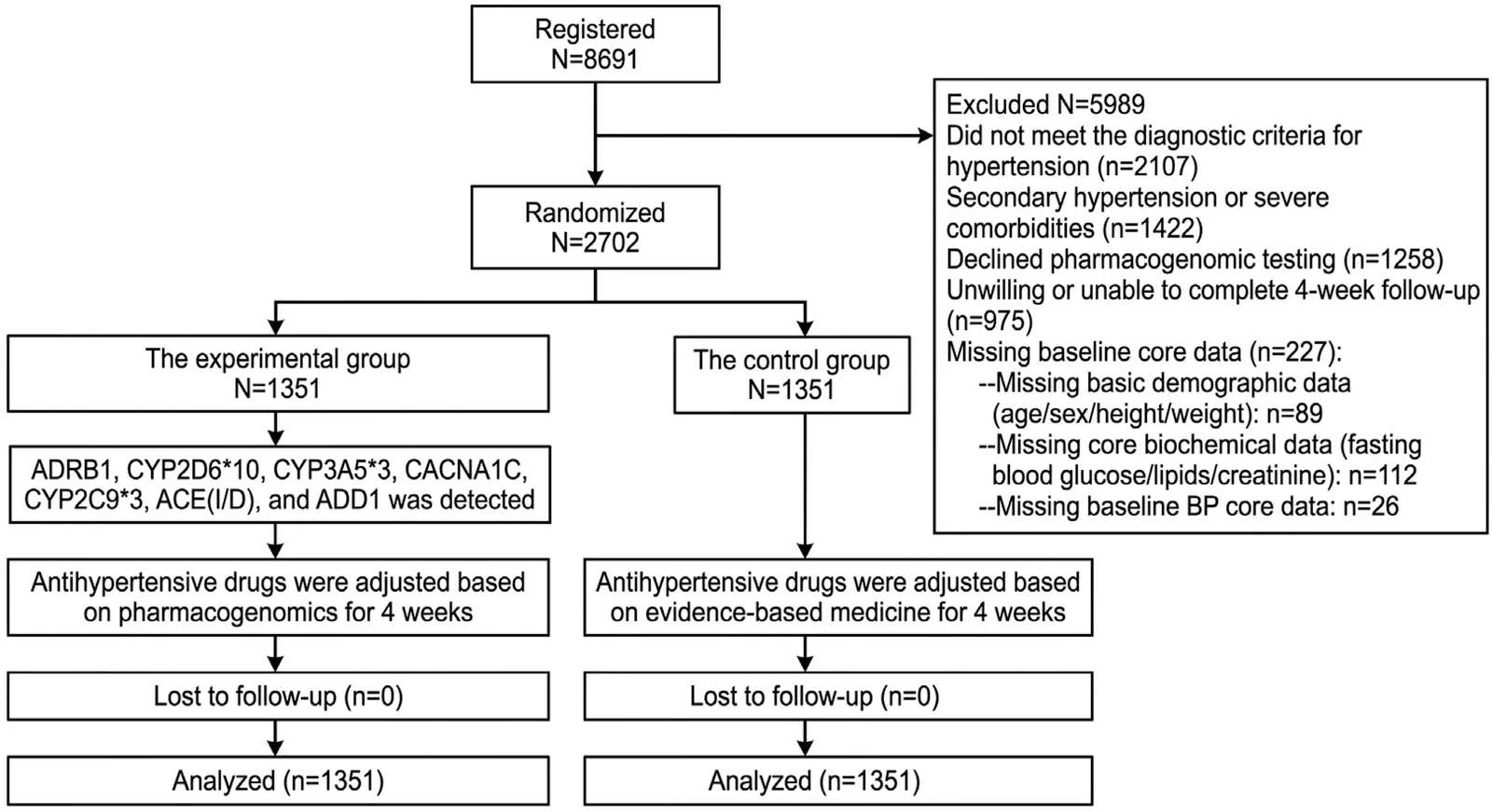
Flowchart of patient participation in the study.

#### Randomization and allocation concealment

Eligible participants were randomly assigned in a 1:1 ratio to either the control group or the experimental group. Randomization was stratified by participating center to ensure balance across sites. The allocation sequence was generated by an independent statistician who was not involved in patient recruitment or outcome assessment, using SAS version 9.4 with a permuted block design (block size of 4). Allocation concealment was maintained using sequentially numbered, opaque, sealed envelopes. These envelopes were prepared by the same statistician and stored in a central location. When a patient was deemed eligible and had completed all baseline assessments, the local investigator opened the next envelope in sequence to reveal the group assignment. Thus, allocation concealment was preserved until the moment of randomization. Participants, treating physicians, and outcome assessors were not blinded to group assignment due to the open-label design (as stated in the title).

### Intervention

For participants in the experimental group, antihypertensive treatment decisions were made by integrating pharmacogenomic results with clinical parameters, including baseline blood pressure, comorbidities (diabetes, chronic kidney disease, cardiovascular disease), concomitant medications (e.g., NSAIDs, potassium-sparing diuretics), and renal function. All attending cardiologists at the participating centers received standardized training on the interpretation of pharmacogenomic reports and the study protocol. The pharmacogenomic report provided genotype-specific recommendations, and physicians followed the precautionary notes embedded in the report, such as avoiding RAAS dual blockade (ARB + ACEI or aliskiren) in patients with diabetes or reduced GFR (<60 mL/min), monitoring serum potassium when using potassium-sparing agents, and being aware that NSAIDs may attenuate the antihypertensive effect of ARBs/ACEIs. The pharmacogenomics-guided treatment algorithm was predefined as follows. For *ADRB1* (Arg389Gly) and *CYP2D6*10, patients carrying the *ADRB1* C allele (Arg389), which is associated with enhanced β-blocker response, were preferentially prescribed a β-blocker (metoprolol). In *CYP2D6*10 carriers (reduced metabolizer phenotype), the starting dose of metoprolol was reduced by 50% (e.g., 12.5 mg twice daily instead of 25 mg). For the *ACE* I/D polymorphism, no dose adjustment was recommended based on our negative association analysis (Supplementary Table S2). For *CYP2C9*3, carriers of the *CYP2C9*3 allele were advised to avoid losartan (due to reduced activation) or to use an alternative ARB such as irbesartan, which is less dependent on CYP2C9 metabolism. Regarding *CYP3A5*3 and *CACNA1C*, calcium channel blockers (CCBs) were not used as monotherapy in *CACNA1C* G allele carriers; instead, an ARB or ACEI was added preferentially. The *CYP3A5*3 genotype did not alter amlodipine dosing in this cohort. For *ADD1* (Gly460Trp), thiazide diuretics were avoided as first-line monotherapy in *ADD1* T allele carriers due to predicted reduced response; a CCB or ARB was preferred instead. The complete genotype-drug pairing matrix and dose adjustment recommendations are summarized in Supplementary Table S1 (pharmacogenomic report template). Physicians followed these predefined rules when initiating or adjusting therapy. Combination therapy was started according to the 2010 Chinese hypertension guideline (see above), but the choice of drug classes was guided by the genotype profile. The choice between monotherapy and combination therapy followed the 2010 Chinese hypertension guideline: monotherapy was initiated for patients with baseline SBP <160 mmHg and DBP <100 mmHg without high-risk conditions; combination therapy (preferably ACEI/ARB + CCB or ACEI/ARB + thiazide) was started when baseline SBP ≥160 mmHg and/or DBP ≥100 mmHg, or in patients with diabetes, chronic kidney disease, cardiovascular disease, or target organ damage even if BP was in the 140–159/90–99 mmHg range.

### Follow-up

All participants were followed for 4 weeks. Assessments were performed at baseline (week 0) and at the end of the 4-week treatment period (week 4); no interim visits were scheduled. A standardized case report form (CRF; see Supplementary Material) was used to prospectively record the following parameters: seated office systolic/diastolic blood pressure and heart rate; predefined adverse drug reactions for each drug class (e.g., dry cough, hyperkalemia, bradycardia, ankle edema, electrolyte disturbances, hyperuricemia); current antihypertensive regimen (drug name, dose, frequency) to assess adherence; biochemical parameters (fasting glucose, uric acid, creatinine, alanine aminotransferase, creatine kinase, serum potassium, sodium, and lipid profile); electrocardiogram findings; and lifestyle factors (smoking, alcohol, high-salt diet, physical inactivity, psychological stress). Data were entered by trained research coordinators, and double data entry was performed to ensure accuracy. Loss-to-follow-up rates did not differ significantly between the two groups (see Results).

### Genetic analysis

Pharmacogenomic testing was performed only for participants in the experimental group (*n* = 1,351), as genetic information was required to guide their antihypertensive treatment. The control group (*n* = 1,351) received standard care without genotyping.

For each participant, 10 *μ*L of peripheral whole blood was treated with a nucleic acid releasing agent. Then, 1 μL of the processed sample was added to the amplification mixture. The targeted SNP loci were amplified using a commercial fluorescence PCR kit (Jinan Guangyin Company, China). Subsequently, genotyping of seven selected single nucleotide polymorphism loci—ACE (I/D, rs1799752), CYP2C93 (c.1075A > C, rs1057910), ADRB1 (c.1165G > C, rs1801253, Arg389Gly), CYP2D610 (c.100C > T, rs1065852), CYP3A53 (c.219-237A > G, rs776746), CACNA1C (c.50-1658T > G, rs2238032), and ADD1 (c.1378G > T, rs4961, Gly460Trp)—was performed using the TaqMan-MGB probe method. PCR amplification was carried out on a fluorescence quantitative PCR detection system (FQD-48A, Hangzhou Bioer Company, China) following the manufacturer's instructions. Genotype determination was based on fluorescence signal and amplification curve profiles.

Quality control samples were analyzed in each run to monitor the validity of the genotyping results. Positive and negative controls were included in every assay to monitor the entire TaqMan-MGB probe-based genotyping system, thereby eliminating potential experimental interferences and ensuring accurate genotyping. Additionally, approximately 5% of randomly selected samples were re-genotyped for quality assurance, with 100% concordance.

## Clinical data collection

We collected blood genetic samples from all 2,702 hypertensive patients to analyze genetic variants across seven key gene loci: *ACE* (I/D, rs1799752), *CYP2C9* (*3, c.1075A > C, rs1057910), *ADRB1* (c.1165G > C, rs1801253, Arg389Gly), *CYP2D6* (*10, c.100C > T, rs1065852), *CYP3A5* (*3, c.219-237A > G, rs776746), *CACNA1C* (c.50-1658T > G, rs2238032), and *ADD1* (c.1378G > T, rs4961, Gly460Trp). These genetic variations are linked to the metabolism, transport, and therapeutic targets of five frontline antihypertensive drug classes—angiotensin-converting enzyme inhibitors (ACEIs), angiotensin II receptor blockers (ARBs), beta-blockers, calcium channel blockers (CCBs), and diuretics. The presence of these genetic variants is known to influence the effectiveness of antihypertensive treatments. Prior to and following the treatment period, all participants were subjected to blood pressure measurements and a comprehensive biochemical assessment. This included measurements of total cholesterol (TC), triglycerides (TG), high-density lipoprotein cholesterol (HDL-C), low-density lipoprotein cholesterol (LDL-C), fasting blood glucose, potassium, sodium, serum creatinine, serum uric acid, and alanine aminotransferase (ALT) levels to monitor the impact of the treatment on these critical health indicators. All clinical and biochemical data were extracted from the hospital's electronic medical record (EMR) system and laboratory information system (LIS) at each participating center. Baseline information including demographics, medical history, and vital signs were retrieved from the EMR, while biochemical parameters (e.g., fasting blood glucose, lipid profiles, serum creatinine, uric acid, electrolytes, and ALT) were obtained from the LIS. Data collection was performed by trained research coordinators using a standardized case report form, and double data entry was applied to ensure accuracy.

## Office blood pressure measurement

To ensure accurate blood pressure readings, participants were instructed to sit quietly for a minimum of five minutes in a chair, with their arm cuff positioned at heart level. They were also advised to abstain from smoking for at least 30 min prior to the measurement. Blood pressure was measured multiple times, with each measurement taken consecutively and separated by an interval of at least one minute. The procedural protocol allowed for the calculation of an average blood pressure from the two closest readings, which was then recorded as the participant's seated blood pressure.

## Primary and secondary outcome measures

The study's primary outcome measure is the reduction in blood pressure observed after a 4-week intervention period from the study's commencement. This key indicator is crucial for assessing the immediate efficacy of the treatment regimens provided.

Additionally, we have identified a range of secondary outcome measures to evaluate the broader impact of the treatments on cardiovascular health. These include changes in the following parameters after the 4-week treatment period: TG, TC, LDL-C, HDL-C, fasting blood glucose, potassium levels, sodium levels, serum creatinine, serum uric acid, and ALT. These secondary measures provide a comprehensive assessment of the treatment's influence on metabolic and renal function, which are critical factors in managing hypertension and associated comorbidities.

### Sample size calculation

The sample size was determined based on an expected difference in systolic blood pressure reduction of 5 mmHg between the two groups, with a standard deviation of 15 mmHg, a two-sided significance level (*α*) of 0.05, and a power (1 − *β*) of 90%. Assuming a 10% dropout rate, a minimum of 1,350 participants per group (2,700 total) was required. The actual enrollment of 2,702 participants met this requirement.

## Multi-genetic variant burden analysis

To evaluate the cumulative effect of genetic variants on treatment response, a risk allele burden score was calculated for each patient in the experimental group. Risk alleles were predefined based on published evidence and as indicated in Table 2: *ACE* D, *CYP2C9* *3, *ADRB1* C, *CYP2D6* *10, *CYP3A5* *3, *CACNA1C* G, and *ADD1* T. The total number of risk alleles per patient (range 0–14) was summed across the seven loci. Patients were then categorized into three groups: 0 variants, 1 variant, and ≥2 variants. One-way ANOVA was used to compare blood pressure reduction among groups, and the chi-square test was used to compare blood pressure control rates and regimen change rates. Statistical significance was set at *P* < 0.05.

## Statistical analysis

The normality of the quantitative data was initially assessed using the Kolmogorov–Smirnov test. Data adhering to a normal distribution were expressed as the mean ± standard deviation, and differences between the two groups were compared using the independent samples *t*-test. In contrast, data not conforming to a normal distribution were reported as medians with interquartile ranges, and group differences were compared using the Mann–Whitney *U* test. Categorical data were presented as percentages. To evaluate the changes within each group over the course of the study, paired McNemar's tests were utilized to compare the use of antihypertensive medications before and after the intervention. Between-group comparisons for categorical outcomes were conducted using the chi-square (*χ*²) test. Furthermore, analysis of covariance (ANCOVA) was applied to compare blood pressure changes between the groups, with adjustments for potential confounding factors such as age, gender, and body mass index (BMI). Statistical significance was set at a *p*-value of less than 0.05. All statistical analyses were conducted using SPSS software, version 26.0 (IBM Corp., Armonk, NY, USA), ensuring robust and reliable computational processing.

## Results

### Baseline characteristics

The study population comprised 2,702 individuals with hypertension, evenly distributed into two groups. The experimental group consisted of 780 males with a mean age of 59.97 ± 12.72 years, while the control group included 756 males with a mean age of 61.48 ± 11.36 years. At the outset, both groups exhibited comparable baseline characteristics regarding gender distribution, BMI, prevalence of CVD, and hyperlipidemia. The experimental group had a significantly higher prevalence of diabetes (1.2% vs. 0.1%, *P* < 0.001) and stroke (0.5% vs. 0.1%, *P* = 0.034) compared with the control group. Small but statistically significant differences were also observed in fasting blood glucose (6.23 ± 2.02 vs. 6.55 ± 2.22 mmol/L, *P* < 0.001), total cholesterol (4.93 ± 1.19 vs. 4.62 ± 1.24 mmol/L, *P* < 0.001), LDL-C (2.81 ± 0.93 vs. 2.59 ± 0.91 mmol/L, *P* < 0.001), and uric acid (341.94 ± 97.49 vs. 324.07 ± 96.25 μmol/L, *P* < 0.001). In contrast, no significant differences were found in triglycerides (*P* = 0.465) or HDL-C (*P* = 0.185). The absolute differences in these parameters were small, indicating broadly similar baseline health status between the two groups (Table 1).

**Table 1:** Baseline characteristics of participants in the study.

Variables	All (*n* = 2,702)	Control group (*n* = 1,351)	Experimental group (*n* = 1,351)	*P* value
Age (years)	60.72 ± 12.08	61.48 ± 11.36	59.97 ± 12.72	0.002
Male, *n* (%)	1,536 (56.7%)	756 (56%)	780 (57.7%)	0.351
Diabetes, *n* (%)	17 (0.7%)	1 (0.1%)	16 (1.2%)	<0.001
Stroke, *n* (%)	8 (0.3%)	1 (0.1%)	7 (0.5%)	0.034
CVD, *n* (%)	36 (1.3%)	14 (1%)	22 (1.7%)	0.179
Hyperlipidemia	15 (0.6%)	4 (0.3%)	11 (0.8%)	0.07
BMI (kg/m^2^)	24.89 ± 3.28	24.83 ± 3.31	24.96 ± 3.24	0.521
SBP (mmHg)	155.99 ± 11.71	156.52 ± 11.46	155.45 ± 11.93	0.211
DBP (mmHg)	91.93 ± 10.68	91.75 ± 10.27	92.12 ± 11.08	0.186
FBS (mmol/L)	6.39 ± 2.13	6.55 ± 2.22	6.23 ± 2.02	<0.001
SUA (mmol/L)	333.01 ± 97.27	324.07 ± 96.25	341.94 ± 97.49	<0.001
Scr (μmol/L)	71 (59, 86)	70 (58, 86)	72 (60, 86)	0.087
ALT (U/L)	22.3 (16, 32.1)	22.6 (16, 33)	22 (15.4, 31.2)	0.049
Potassium (mmol/L)	3.97 ± 0.41	3.96 ± 0.41	3.97 ± 0.42	0.044
Sodium (mmol/L)	139.99 ± 3.97	140.08 ± 4.37	139.91 ± 3.53	<0.001
TC (mmol/L)	4.78 ± 1.22	4.62 ± 1.24	4.93 ± 1.19	<0.001
TG (mmol/L)	1.58 (1.15, 2.34)	1.57 (1.17, 2.3)	1.6 (1.14, 2.41)	0.465
HDL-C (mmol/L)	1.31 ± 0.42	1.31 ± 0.45	1.29 ± 0.39	0.185
LDL-C (mmol/L)	2.70 ± 0.93	2.59 ± 0.91	2.81 ± 0.93	<0.001

CVD, cardiovascular disease; BMI, body mass index; SBP, systolic blood pressure; DBP, diastolic blood pressure. FBS, fasting plasma glucose; SUA, serum uric acid; Scr, serum creatinine; ALT, alanine aminotransferase; TC, total cholesterol; TG, triglyceride; HDL-C, high-density; lipoprotein cholesterol; LDL-C, low-density lipoprotein cholesterol.

Normal variables are presented as mean ± standard deviation; Nonnormal variables are presented as median (interquartile ranges). Independent samples *t*-test is used for between-group comparisons. Categorical data are presented as percentages (%). *P*-values for the difference between the experimental and the control groups at baseline were analyzed by *t*-test for parametric variables, Mann–Whitney *U* test for non-parametric, and *χ*2 test for categorical variables.

### Frequency distribution of genetic variants in genes related to antihypertensive drugs

The distribution of genotype frequencies—comprising wild-type homozygotes, heterozygotes, and homozygous variants—of key genes associated with antihypertensive drug response, including *ACE (I/D)*, *CYP2C9*, *ADRB1*, *CYP2D6*, *CYP3A5*, *CACNA1C*, and *ADD1*, is detailed in Table 2. Specifically, the risk allele frequencies were as follows: *ACE* at 34.3%, *CYP2C9* at 4.8%, *ADRB1* at 74.8%, *CYP2D6* at 57.3%, *CYP3A5* at 69.8%, *CACNA1C* at 11.9%, and *ADD1* at 46.3%. These frequencies provide critical insights into the genetic predispositions influencing the efficacy of antihypertensive medications. Furthermore, an analysis of antihypertensive drug utilization patterns before and after the treatment period revealed notable differences between the two study groups. The control group's medication regimen was diversified, encompassing ARBs, beta-blockers, CCBs, and diuretics. Conversely, the experimental group predominantly utilized ARBs and beta-blockers (Table 3a).

**Table 2:** Distribution of genetic variants in antihypertensive drug-related genes.

Gene locus	Genotype (frequency)			Allele frequency	
	Wild-type	Heterozygous	Variant homozygous	Wild	Variant
*ACE* (I/D)	I/I: 598 (44.3%)	I/D: 578 (42.8%)	D/D: 175 (12.9%)	I: 1,774 (65.7%)	D: 928 (34.3%)
CYP2C9 *3	*1/*1: 1,223 (90.5%)	*1/*3: 127 (9.4%)	*3/*3: 1 (0.1%)	*1: 2,573 (95.2%)	*3: 129 (4.8%)
*ADRB1*	G/G: 85 (6.2%)	G/C: 512 (37.8%)	C/C: 754 (55.8%)	G: 682 (25.2%)	C: 2,020 (74.8%)
CYP2D6 *10	*1/*1: 292 (21.6%)	*1/*10: 571 (42.3%)	*10/*10: 488 (36.1%)	*1: 1,155 (42.7%)	*10: 1,547 (57.3%)
CYP3A5*3	*1/*1: 127 (9.4%)	*1/*3: 562 (41.6%)	*3/*3: 662 (49.0%)	*1: 816 (30.2%)	*3: 1,886 (69.8%)
*CACNA1C*	T/T: 1,047 (77.5%)	T/G: 286 (21.2%)	G/G: 18 (1.3%)	T: 2,380 (88.1%)	G: 322 (11.9%)
*ADD1*	G/G: 396 (29.3%)	G/T: 660 (48.9%)	T/T: 295 (21.8%)	G: 1,452 (53.7%)	T: 1,250 (46.3%)

I/D, insertion/deletion; *1, wild-type allele; *3, 10, variant alleles.

*The variant (risk) alleles for altered drug response are: ACE D; CYP2C9 *3; ADRB1 C (Arg); CYP2D6 *10; CYP3A5 3; CACNA1C G; ADD1 T.

Allele frequencies were calculated as the percentage of each allele among all alleles (2 × number of participants).

**Table 3a:** Comparison of antihypertensive drug use before and after treatment.

Antihypertensive drugs	Baseline (*n*)	4 weeks (*n*)	*P* value
Control group (*n* = 1,351)			
ACEIs (*n*)	59	68	0.211
ARBs (*n*)	341	466	<0.001
Beta blockers (*n*)	147	205	<0.001
CCBs (*n*)	855	1,007	<0.001
Diuretics (*n*)	34	47	0.019
Experimental group (*n* = 1,351)			
ACEIs (*n*)	69	64	0.691
ARBs (*n*)	982	369	<0.001
Beta blockers (*n*)	78	201	<0.001
CCBs (*n*)	753	722	0.200
Diuretics (*n*)	43	35	0.332

ACEIs, angiotensin I-converting enzyme inhibitors; ARBs, angiotensin II receptor blockers; CCBs, calcium channel blockers.

The McNemar test is used to compare the proportions of two groups of antihypertensive drugs.

### Changes in treatment patterns

To further understand the mechanisms underlying blood pressure reduction, we first analyzed the distribution of monotherapy vs. combination therapy and treatment stability during the 4-week period. As shown in Table 3b, at baseline the experimental group had a higher proportion of patients not taking any antihypertensive medication (28.5%) compared to the control group (11.6%), while the control group had more patients on monotherapy (72.2% vs. 50.6%). The overall distribution of treatment types at baseline differed significantly between the two groups (*P* < 0.001).

**Table 3b:** Monotherapy vs. combination therapy and treatment stability.

Parameter	Control group (*n* = 1,351)	Experimental group (*n* = 1,351)	*P* value^a^
Baseline (week 0)			
No medication, *n* (%)	156 (11.55%)	385 (28.50%)	<0.001
Monotherapy, *n* (%)	976 (72.24%)	683 (50.56%)	
Combination therapy (≥2 drugs), *n* (%)	219 (16.21%)	283 (20.95%)	
Week 4			
No medication, *n* (%)	19 (1.41%)	26 (1.92%)	<0.001
Monotherapy, *n* (%)	944 (69.87%)	712 (52.70%)	
Combination therapy (≥2 drugs), *n* (%)	388 (28.72%)	613 (45.37%)	
Treatment stability			
Remained on same regimen, *n* (%)	911 (67.43%)	279 (20.65%)	<0.001
Regimen changed, *n* (%)	440 (32.57%)	1,072 (79.35%)	

a *P* values were calculated by chi-square test for the distribution of treatment patterns (no medication/monotherapy/combination) at each time point and for the proportion of regimen changes between the two groups.

At week 4, the proportion of patients on no medication decreased to very low levels in both groups (control 1.4%, experimental 1.9%). The control group remained predominantly on monotherapy (69.9%), with only 28.7% receiving combination therapy. In contrast, the experimental group showed a marked shift toward combination therapy, with 45.4% of patients on two or more drugs, while monotherapy decreased to 52.7% (*P* < 0.001 for between-group comparison at week 4). Notably, 79.4% of patients in the experimental group experienced a change in their antihypertensive regimen during the 4-week period, compared to only 32.6% in the control group (*P* < 0.001). These findings indicate that genotype-guided therapy was associated with significantly more frequent treatment adjustments and a greater adoption of combination therapy, which likely contributed to the superior blood pressure control observed in the experimental group.

### Multi-genetic variant burden analysis

To explore whether the number of risk alleles influences treatment response, we calculated the total number of risk alleles for each patient in the experimental group (*n* = 1,351). Risk alleles were defined as: *ACE* D, *CYP2C9* *3, *ADRB1* C, *CYP2D6* *10, *CYP3A5* *3, *CACNA1C* G, and *ADD1* T. The distribution of risk allele counts is shown in Table 3c. The majority of patients (95.0%) carried two or more risk alleles.

**Table 3c:** Association of multi-genetic variant burden with treatment outcomes in the experimental group.

Variant burden	Patients, *n* (%)	SBP reduction (mmHg)^a^	DBP reduction (mmHg)^a^	BP control rate, *n* (%)^b^
0 variants	3 (0.2%)	−42.7 ± 15.8	−20.0 ± 12.5	3 (100%)
1 variant	64 (4.7%)	−24.9 ± 13.4	−16.3 ± 10.9	58 (90.6%)
≥2 variants	1,284 (95.0%)	−26.5 ± 33.0	−15.1 ± 11.3	1,116 (86.9%)
*P* value		0.64	0.53	0.55

*P* values were calculated using one-way ANOVA for blood pressure reduction and chi-square test for control rate and regimen change rate. Comparisons between the three groups showed no statistically significant differences for any outcome.

a Risk alleles defined as: *ACE* D, *CYP2C9* *3, *ADRB1* C (Arg), *CYP2D6* *10, *CYP3A5* *3, *CACNA1C* G, and *ADD1* T. Values are mean ± standard deviation.

b Blood pressure control defined as seated office SBP <140 mmHg and DBP <90 mmHg at week 4.

Patients were categorized into three groups based on variant burden (0, 1, and ≥2 variants). As summarized in Table 3c, there were no statistically significant differences among the three groups in systolic blood pressure reduction (*P* = 0.64), diastolic blood pressure reduction (*P* = 0.53), blood pressure control rate (*P* = 0.55), or regimen change rate (*P* = 0.53). These exploratory analyses suggest that treatment response to pharmacogenomics—guided therapy was not significantly modified by the number of risk alleles in this short-term study.

We further analyzed the association of each genetic variant with blood pressure reduction, control rate, and regimen change rate in the experimental group (*n* = 1,351). Detailed results are presented in Supplementary Table S2. In brief, *CACNA1C* G allele carriers (*n* = 304) had a significantly smaller reduction in diastolic blood pressure than non-carriers (–13.56 ± 10.74 mmHg vs. −15.62 ± 11.33 mmHg, *P* = 0.004) and a significantly higher rate of regimen changes (85.2% vs. 77.7%, *P* = 0.005; OR = 1.66, 95% CI: 1.17–2.35). Additionally, *ADD1* T allele carriers (*n* = 955) achieved a significantly lower blood pressure control rate than non-carriers (85.8% vs. 90.4%, *P* = 0.026; OR = 0.64, 95% CI: 0.44–0.94). No other variants showed significant associations with any of the outcomes (all *P* > 0.05). These findings support the clinical relevance of *CACNA1C* and *ADD1* genotyping in guiding antihypertensive therapy.

### Changes in blood pressure

Throughout the 4-week treatment period, there was a marked reduction in blood pressure for both the control and experimental groups, with all parameters achieving statistical significance (*P* < 0.001). Specifically, in the control group, SBP and DBP dropped from an initial average of 156.52/91.75 mmHg to 143.74/82.73 mmHg. The experimental group experienced a more pronounced decline, with SBP and DBP levels falling from 155.45/92.12 mmHg to 129.03/76.96 mmHg, as detailed in Table 4. The control group exhibited a decrease in SBP and DBP of −10 (−19, −3) mmHg and −8 (−16, −2) mmHg, respectively, over the 4 weeks. In contrast, the experimental group saw more significant reductions of −27 (−36, −18) mmHg for SBP and −15 (−22, −8) mmHg for DBP, with the between-group differences being statistically significant for both SBP and DBP (*P* < 0.001), as depicted in Figure 3. At the outset, all participants presented with uncontrolled blood pressure. Post-treatment, the control group reached a blood pressure control rate, defined as seated office readings below 140/90 mmHg, of 21.9%. Impressively, the experimental group attained a substantially higher control rate of 87.1% (*P* < 0.001), indicating the superior efficacy of the genotype-guided treatment approach.

**Figure 3:**
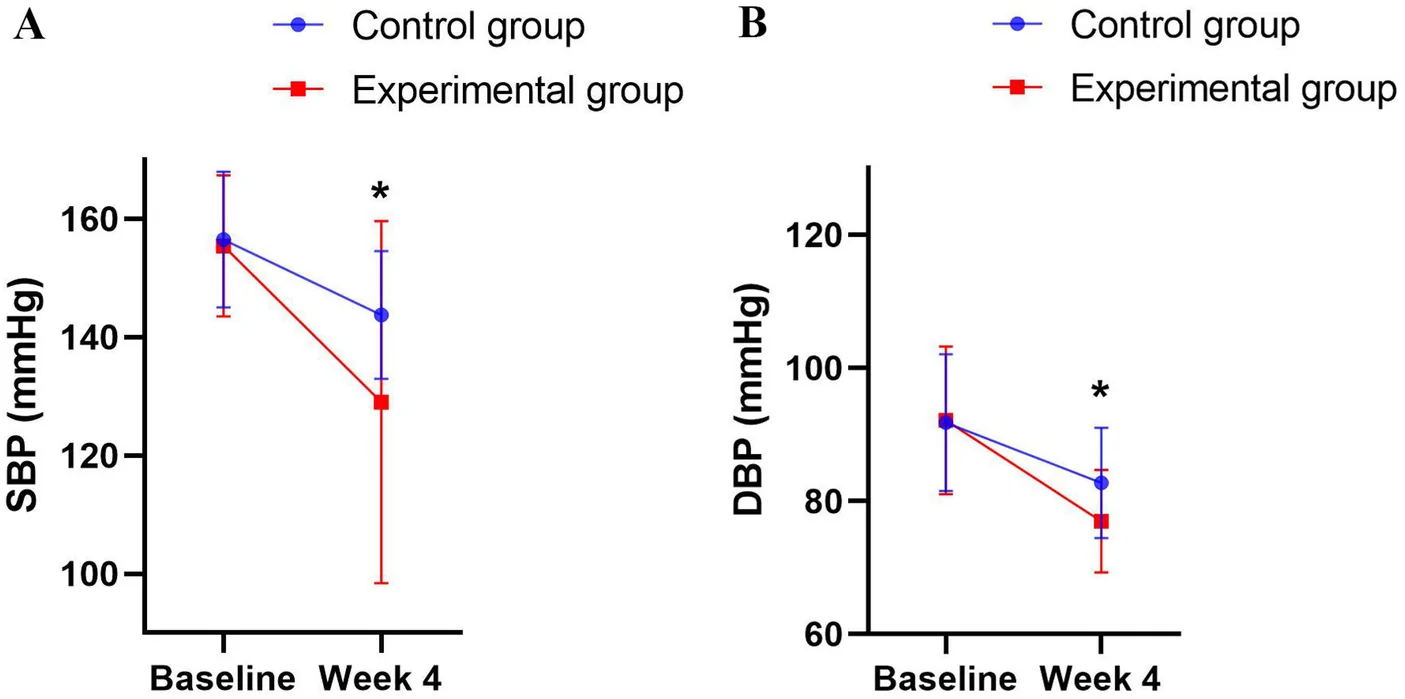
Changes in blood pressure among participants in the study. Changes in systolic blood pressure **(A)** and diastolic blood pressure **(B)** among participants in the study. *, *p* < 0.05.

**Table 4:** Changes in BP and rate of achievement of target BP among participants in the study.

Variable	Control (*n* = 1,351)	Experimental group (*n* = 1,351)	*P* value
BP level, mmHg			
Seated office SBP			
Baseline	156.52 ± 11.46	155.45 ± 11.93	0.211
4 weeks	143.74 ± 10.84	129.03 ± 30.60	<0.001
Change between weeks 0 and 4	−10(−19, −3)	−27(−36, −18)	<0.001
Seated office DBP			
Baseline	91.75 ± 10.27	92.12 ± 11.08	0.186
4 weeks	82.73 ± 8.28	76.96 ± 7.71	<0.001
Change between weeks 0 and 4	−8 (−16, −2)	−15 (−22, −8)	<0.001
Rate of achievement target BP, %			
Seated office BP (<140/90 mmHg)			
4 weeks	297 (21.9%)	1,177 (87.1%)	<0.001

BP, blood pressure; SBP, systolic blood pressure; DBP, diastolic blood pressure.

Change shows a reduction (%) between baseline and 4 weeks. Analysis of covariance (ANCOVA) is used to compare blood pressure differences between groups after adjusting for age, gender, and BMI, *χ*^2^ test is used to compare the rate of achievement target blood pressure between two groups.

### Changes in secondary endpoints

As presented in Table 5, the study's secondary endpoints were monitored to assess the broader health impacts of the treatment. Changes in secondary endpoints are summarized in Table 5. No significant differences were observed between the two groups in the change of serum creatinine (*P* = 0.231), serum potassium (*P* = 0.225), or triglycerides (*P* = 0.466). In contrast, the experimental group showed significantly greater reductions than the control group in serum uric acid (*P* = 0.001), total cholesterol (*P* < 0.001), and LDL-C (*P* < 0.001). Fasting blood glucose also decreased more in the control group (*P* < 0.001), and serum sodium decreased significantly more in the control group than in the experimental group (*P* < 0.001). Alanine aminotransferase (ALT) changes also differed significantly between groups (*P* = 0.003).

**Table 5:** Changes in secondary endpoints Among participants in the study.

Variable	Control group (*n* = 1,351)	Experimental group (*n* = 1,351)	*P* value
FBS (mmol/L)	−0.3 (−0.98, −0.04)	−0.24 (−0.8, 0.03)	<0.001
SUA (mmol/L)	−5.8 (−25, 6)	−8.4 (−34.7, 3.4)	0.001
Scr (μmol/L)	−1.26 (−5.6, 4)	−1.6 (−6.6, 3.4)	0.231
ALT (U/L)	−0.6 (−3, 3.3)	0.6(−3, 4)	0.003
Potassium (mmol/L)	0.01 (−0.2, 0.21)	0.03 (−0.12, 0.19)	0.225
Sodium (mmol/L)	−0.6 (−2.5, 1.7)	−0.1 (−1.7, 1.6)	<0.001
TC (mmol/L)	−0.07 (−0.33, 0.14)	−0.18 (−0.56, 0.02)	<0.001
TG (mmol/L)	−0.04 (−0.24, 0.08)	−0.05 (−0.25, 0.06)	0.466
LDL-C (mmol/L)	−0.07 (−0.34, 0.1)	−0.17 (−0.5, 0.01)	<0.001
HDL-C (mmol/L)	0 (−0.08, 0.14)	−0.01 (−0.11, 0.09)	<0.001

FBS, fasting plasma glucose; SUA, serum uric acid; Scr, serum creatinine; ALT, alanine aminotransferase; TC, total cholesterol; TG, triglyceride; LDL-C, low-density lipoprotein cholesterol; HDL-C, high-density lipoprotein cholesterol; LDL-C, low-density lipoprotein cholesterol.

Change shows a reduction (%) between baseline and 4 weeks. *P*-values for the difference between the two groups at baseline were analyzed by Mann–Whitney *U* test.

### Adverse events

Adverse events were recorded in both groups during the 4-week period. In the experimental group, 4 patients (0.3%) reported adverse events, including dry cough (*n* = 2, 0.15%), neurotic edema (*n* = 1, 0.07%), and dizziness (*n* = 1, 0.07%). No adverse events were reported in the control group. No serious adverse events (e.g., hospitalization, life-threatening events, death) occurred in either group. There were no significant differences between the two groups in any specific adverse event or in the overall adverse event rate (all *P* > 0.05, Fisher's exact test). Detailed safety data are presented in Supplementary Table S10.

## Discussion

In this multi-center randomized controlled trial, we explored the potential of pharmacogenomics to enhance personalized treatment for uncontrolled primary hypertension. Our findings reveal a significant and substantial decrease in blood pressure in both the experimental and control groups following the medication intervention. Notably, the experimental group, which was guided by pharmacogenomic insights, exhibited a more pronounced reduction in blood pressure and achieved a higher rate of blood pressure control compared to the control group. However, it is important to note that the observed effect size represents short-term maximal efficacy achieved under intensive, open-label management, rather than the expected long-term outcome in routine clinical practice. These compelling results substantiate the safety and reliability of pharmacogenomics-guided medication selection in the treatment of hypertension.

Hypertension stands as a critical modifiable risk factor for cardiovascular diseases, yet globally, its management often falls short of optimal standards. The current paradigm for selecting antihypertensive drugs is predominantly informed by clinical experience and established hypertension guidelines. Despite a plethora of available medications, the challenge of identifying efficacious drugs with minimal side effects for individual patients persists, as does the challenge of reducing their economic and cardiovascular risks. The variability in patients' responses to these drugs is predominantly influenced by genetic factors, which complicates the task of clinicians who rely solely on experience to make accurate medication choices. However, the landscape is shifting with the rise of personalized treatment approaches for hypertension. The advent of omics technologies, particularly pharmacogenomics, has laid a robust foundation for precision medicine. By providing genetic insights into key enzymes that govern drug metabolism, transport, and target engagement, pharmacogenomics is emerging as a pivotal tool to enhance the precision of antihypertensive drug selection.

The adoption of pharmacogenomics to guide the selection of antihypertensive drugs represents a burgeoning therapeutic strategy that has begun to reveal its potential through initial clinical inquiries. For instance, a clinical cohort study that encompassed 59 patients with resistant hypertension (RH) was instrumental in assessing the efficacy and safety of pharmacogenomics-tailored treatment protocols. Patients with RH were randomly allocated to either an intervention group, which received personalized pharmacogenomics-guided treatment, or a control group, which adhered to conventional consensus treatment, over a period of 12 weeks. The findings were striking: the intervention group exhibited a significantly greater reduction in both systolic (31.26 ± 18.64 mmHg vs. 14.61 ± 17.74 mmHg; *p* = 0.001) and diastolic blood pressure (19.61 ± 17.32 mmHg vs. 7.81 ± 11.23 mmHg; *p* = 0.003) compared to the control group (10). Furthermore, a comparative analysis within a Chinese population underscored the advantages of genotype-guided treatment over traditional clinically experienced-guided antihypertensive therapy. The genotype-guided group not only demonstrated significant reductions in systolic and diastolic blood pressure but also achieved a remarkable increase in the blood pressure control rate, outperforming the clinically guided group with a notable margin (8.5% vs. 3.6%, *p* < 0.01). This disparity was particularly pronounced among individuals with stage 2 and 3 hypertension, highlighting the value of personalized medicine in optimizing treatment outcomes (26).

The greater blood pressure reduction in the experimental group was accompanied by substantially different treatment patterns (Table 3b). The experimental group had a much higher rate of regimen changes (79.4% vs. 32.6%) and a larger increase in combination therapy (from 20.9% to 45.4%) compared with the control group (from 16.2% to 28.7%). These differences indicate that pharmacogenomic guidance prompted clinicians to adjust treatment more actively, particularly by adding a second drug when genetic variants predicted reduced response to initial monotherapy (e.g., *CACNA1C* G allele for CCBs, *ADD1* T allele for thiazides). This more frequent and rational use of combination therapy is the most likely explanation for the superior blood pressure control observed in the experimental group. Detailed subgroup, sensitivity, and comparative analyses are provided in Supplementary Tables S3–S8.

Individualized antihypertensive therapy is increasingly reliant on pharmacogenomics to illuminate the genetic variations that influence the activity of metabolizing enzymes, transporters, and receptors pertinent to antihypertensive medications. A case in point is the renin-angiotensin system, which is intimately connected to hypertension and has been a focal point for research, particularly regarding the *ACE* insertion/deletion (I/D) variant. Evidence suggests that the D allele of this polymorphism is correlated with heightened ACE activity across both Caucasian and Asian populations (11, 12, 27), and is also implicated in cardiovascular diseases (28, 29). This association has been mechanistically explained by recent findings that the 287-bp insertion element codes for an AluYa5 RNA that downregulates ACE gene expression, resulting in lower ACE activity in I allele carriers (30). Consequently, the D allele has been linked to improved blood pressure control with ACE inhibitors in some Asian populations (13–15). However, the clinical utility of this genetic marker remains debated. A computational model of blood pressure regulation predicted that a single genetic variant like *ACE* I/D accounts for only a small fraction of heredity in treatment success, and its predictive value is therefore limited (31). Moreover, large-scale clinical trials have failed to demonstrate a consistent association. In the LIFE pharmacogenetics substudy involving 3,503 hypertensive patients, the *ACE* I/D polymorphism did not affect blood pressure reduction or cardiovascular outcomes in patients treated with losartan or atenolol (32). Similarly, a study of 148 Chinese hypertensive patients found no significant association between *ACE* I/D genotype and blood pressure response to telmisartan (33). In our own cohort, the prevalence of the *ACE* D allele was 34.3%, but subsequent analysis showed no significant association between ACE genotype and blood pressure reduction, control rate, or regimen change (all *P* > 0.05). Therefore, routine ACE inhibitor dosing in this population does not appear to require genotype-guided adjustment based solely on the I/D polymorphism.

The *CYP2C9* enzyme, responsible for the metabolism of losartan to its active form E-3174, is another example. The *CYP2C9*2* and *3* alleles are known to diminish enzyme activity, potentially compromising the antihypertensive efficacy of the drug (16). A mathematical modeling study predicted that individuals with the *CYP2C9*1/*1* genotype respond better to losartan therapy than those carrying *2* or *3* alleles (34). This prediction has been supported by an observational study of 81 hypertensive patients, where carriage of *CYP2C9*2* or *3* alleles reduced the hypotensive effect of losartan (p < 0.001, OR = 8.13) (35). A systematic review and meta-analysis further confirmed that *CYP2C9*2* or *3* carriers have significantly altered pharmacokinetics: higher losartan area under the curve (AUC), lower E-3174 AUC, and longer half-lives of both losartan and its metabolite (36). However, evidence from clinical outcome studies is not entirely consistent. In the double-blind, cross-over GENRES study involving 217 hypertensive Finnish men, the CYP2C9*2 or *3* alleles did not influence the antihypertensive effect of losartan in patients with normal kidney function (37). Interestingly, that study also found that the CYP2C9*3 allele was associated with lower baseline plasma renin activity and aldosterone levels, suggesting a more efficient sodium reabsorption capacity that might offset the pharmacokinetic differences.

In our Hunan-based Chinese population, the CYP2C9*3 allele frequency was 4.8%. Our subsequent analysis showed no significant association between CYP2C9*3 genotype and blood pressure response or control rate (all *P* > 0.05). Therefore, while the low frequency of the loss-of-function allele suggests that most patients would metabolize losartan normally, the actual efficacy of losartan in this cohort was not genotype-dependent. This is consistent with the neutral finding from the GENRES study and supports the use of losartan without mandatory genotyping in our population.

The *ADRB1* gene, which encodes the β1-adrenergic receptor—the primary target of β-blockers—exhibits polymorphisms that can impact receptor signaling (18). Variants such as rs1801252 (Ser49Gly) and rs1801253 (Arg389Gly) have been investigated for their influence on treatment outcomes (19). A study in Chinese hypertensive patients reported that the *ADRB1* 1165G > C (Arg389Gly) polymorphism significantly affected the blood pressure response to metoprolol, with Arg389 (C allele) carriers showing greater reductions (20). However, results are not entirely consistent across drug classes or populations. A 2022 study in 99 Chinese hypertensive patients receiving bisoprolol found no significant association between *ADRB1* Arg389Gly and either blood pressure or heart rate response (38). In our Hunan-based Chinese population, the frequency of the *ADRB1* 389Arg-encoding C allele was 74.8%, which is consistent with previous reports in Han Chinese. However, our own analysis showed no significant association between *ADRB1* genotype and blood pressure reduction or control rate (all *P* > 0.05). Therefore, routine genotyping of *ADRB1* for β-blocker selection cannot be recommended based solely on frequency data; clinical decisions should continue to be guided by standard indications and patient response.

The cytochrome P450 3A5 (CYP3A5) enzyme, which plays a crucial role in the hepatic metabolism of calcium channel blockers, is influenced by genetic variants that can significantly affect individual responses to antihypertensive medications (21, 39). This enzyme is also expressed in the kidney and has been implicated in renal sodium reabsorption and blood pressure regulation, with its expression and activity strongly linked to the CYP3A5*3 polymorphism (6986G > A). Specifically, appreciable expression is found in carriers of the CYP3A5*1 (6986A) allele but not in homozygous carriers of the CYP3A5*3 (6986G) allele (40). Notably, investigations by Zhang et al. and Huang et al. have demonstrated a positive correlation between the CYP3A5*3 allele and an enhanced antihypertensive response to amlodipine among Chinese populations (41, 42). In our study, the CYP3A5*3 allele was present in 69.8% of the Hunan regional population, a frequency consistent with recent large-scale reports in Han Chinese hypertensive patients (43). However, in our cohort, we did not find a significant association between CYP3A5*3 genotype and amlodipine-related outcomes (all *P* > 0.05). This negative finding aligns with a recent large-scale UK Biobank study (*N* = 487,171), which concluded that *CYP3A5* variation likely has no meaningful impact on blood pressure-related phenotypes in a general population, with no significant associations observed in the East Asian subgroup (44). Collectively, these data suggest that while CYP3A5*3 is highly prevalent in Han Chinese and has well-established pharmacokinetic effects, its clinical utility for guiding amlodipine therapy remains unproven.

Furthermore, the voltage-gated calcium channel *α*1C (CACNA1C), a key determinant of blood pressure response to calcium channel blockers, exhibits genetic variations that can substantially affect treatment outcomes (23). Early studies identified three *CACNA1C* variants (rs22368032, rs2239050, and rs1051375) that significantly alter responsiveness to amlodipine and felodipine, with specific genotypes being notably associated with uncontrolled hypertension (45, 46). More recent investigations have reinforced this association: a 2024 study in 400 hypertensive patients reported that *CACNA1C* rs2239050 GG genotype carriers had significantly better blood pressure response to amlodipine (*P* = 0.004) (47), and a 2025 study in 133 patients confirmed that the same GG genotype is associated with superior systolic and diastolic blood pressure control (48). Moreover, the INVEST-GENES sub-study (*n* ≈ 1,900) demonstrated that *CACNA1C* rs1051375 genotype modifies cardiovascular outcomes in response to verapamil vs. atenolol, with A/A homozygotes experiencing a 46% reduction in major adverse events (49). In our Hunan-based population, 88.1% of patients carried the *CACNA1C* wild-type (T/T) genotype at rs2238032, but our analysis showed that G allele carriers had a significantly smaller reduction in diastolic blood pressure (*P* = 0.004) and a higher rate of regimen changes (*P* = 0.005). This observation aligns with the directionality reported in the literature (i.e., certain *CACNA1C* alleles predict poorer response to CCB monotherapy) and underscores that even a single nucleotide change can influence clinical outcomes.

The *ADD1* gene encodes *α*-adducin, a cytoskeletal protein involved in renal sodium handling (24). The Gly460Trp (rs4961) polymorphism has been associated with enhanced blood pressure response to hydrochlorothiazide (HCTZ) in some studies, particularly when combined with the ACE I allele (25, 50). A meta-analysis of 1,001 patients confirmed a significant overall association between ADD1 genotype and HCTZ-induced blood pressure reduction (51). However, a large Chinese population meta-analysis found no significant association between ADD1 G460 T and hypertension risk overall, suggesting limited independent clinical utility in this ethnic group (52). Moreover, physiological studies have shown that ADD1 interacts with the WNK1-NEDD4L pathway, where the combined presence of specific variants produces consistent effects on renal Na⁺ handling (*P* = 0.009) and thiazide-induced blood pressure lowering (*P* = 0.008) (53). In our Hunan-based population, the *ADD1* T (460Trp) allele frequency was 46.3%. We observed that T allele carriers had a significantly lower blood pressure control rate (85.8% vs. 90.4%, *P* = 0.026), which appears contrary to the direction reported in some early studies. This discrepancy may reflect the multifactorial nature of thiazide response, where the benefit of the Trp allele may be unmasked only in the presence of specific co-variants or under different dietary salt conditions (53). Consequently, while ADD1 genotyping may provide useful information in certain contexts, its routine use as a standalone biomarker for guiding HCTZ therapy requires further validation, particularly in Chinese populations.

Our study distinguishes itself from prior research in several notable ways. Firstly, we boast a larger sample size, which enhances the statistical power and generalizability of our findings. Secondly, our research is one of the few multicenter trials, providing a more comprehensive and representative insight into the Hunan region's demographic and clinical nuances. Lastly, our findings contribute valuable evidence to guide the selection of antihypertensive medications specifically tailored to the Hunan population. Future studies with longer follow-up (≥6–12 months) are needed to determine whether the observed short-term benefits translate into sustained blood pressure control and reduced cardiovascular events, and to further evaluate the long-term safety of genotype-guided therapy.

## Limitations

Several limitations of this study should be acknowledged. First, because genotyping was performed only in the experimental group (as required for treatment guidance), we cannot directly compare genotype distributions between the two groups. However, as discussed above, randomization and the large sample size ensure that any systematic genetic imbalance is highly unlikely. Second, the open-label design introduced performance bias, as neither participants, treating physicians, nor outcome assessors were blinded to group allocation. This may have led to more frequent follow-up, a lower threshold for treatment intensification, and potentially better adherence in the experimental group, which could have inflated the observed blood pressure differences. However, blood pressure was measured using a standardized protocol with automated devices, and the primary outcome (office BP) is objective; thus, detection bias is less likely to have affected the findings. Nevertheless, our results should be interpreted as the short-term maximal efficacy under intensive, open-label management rather than the expected long-term benefit in a blinded trial or routine practice. Third, the follow-up duration was only 4 weeks, which limits assessment of long-term outcomes and medication adherence. Fourth, the baseline imbalance in untreated patients (28.5% in experimental vs. 11.6% in control) may have contributed to a larger initial response in the experimental group, but the significant difference in blood pressure control persisted after adjusting for baseline covariates. Finally, the exploratory analyses of multi-variant burden (Table 3c) should be interpreted with caution and require validation in independent cohorts. Sixth, although 69% of registered patients were excluded, baseline characteristics were well balanced between included and excluded patients (all SMDs < 0.1, Supplementary Table S9), indicating no clinically meaningful selection bias.

## Conclusion

Our study has demonstrated that pharmacogenomics-guided selection of antihypertensive drugs can lead to a significant reduction in both systolic and diastolic blood pressure, enhance hypertension control rates, and minimize adverse drug reactions. Within the Han Chinese population of Hunan, we observed that the frequency of genetic variants in genes related to antihypertensive drug response, with the exception of *CACNA1C* and *ADD1*, aligned with previous findings. Notably, our analysis revealed a low frequency of the CYP2C9*3 genetic variants among hypertensive patients in Hunan. In contrast, higher frequencies of genetic variants were observed for *ADRB1* and *CYP3A5*, indicating a potential increased sensitivity of this population to the effects of angiotensin receptor antagonists, β-blockers, and calcium channel blockers, which could inform more precise treatment strategies.

## Data Availability

The original contributions presented in the study are included in the article/Supplementary Material, further inquiries can be directed to the corresponding author.
