# Supplementary material for: Pharmacogenomics-guided therapy for essential hypertension: a multi-center randomized controlled open-label trial
Source: Front Cardiovasc Med. 2026 Sep 18;13:1842597. doi: 10.3389/fcvm.2026.1842597 (PMC13632465; doi:10.3389/fcvm.2026.1842597)
Supplement: Supplementary file 1 [file Datasheet1.pdf]

Supplementary S1 Pharmacogenomic report template

AGEN® 时代基因
致力于个体化健康

个体化用药基因检测报告

一、基本信息

患者信息		样本信息		送检信息	
姓　　名：		样本编号：		送检医院：	
性　　别：		样本类型：	全血	送检科室：	
年　　龄：		采样时间：		门诊/住院号:	
民　　族：		接收日期：		送检医生：	
联系电话：				送检时间：	2024-02-04
临床诊断：					
送检项目：	氨氯地平、硝苯地平 CACNA1C(rs2238032)、CYP3A5*3(rs776746);氯沙坦 CYP2C9*3(rs1057910);厄贝沙坦、缬沙坦 CYP2C9*3(rs1057910);卡托普利、赖诺普利 ACE(rs1799752);依那普利、贝那普利、培哚普利、福辛普利 ACE(rs1799752);氢氯噻嗪 ADD1(rs4961);呋塞米、螺内酯 ADD1(rs4961);美托洛尔、普萘洛尔 ADRB1(rs1801253)、CYP2D6(rs1065852);比索洛尔、阿替洛尔 ADRB1(rs1801253)				
检测方法：	PCR荧光探针法			检测设备：	荧光定量聚合酶链反应（PCR）检测系统

二、检测结果

检测项目	基因位点	参考等位基因	检测结果	结果解释(可能的临床表型)
氨氯地平、硝苯地平	CACNA1C(T>G)	T	TT	疗效正常
	CYP3A5*3(A>G)	*3	*3/*3 (慢代谢型)	
氯沙坦	CYP2C9*3(A>C)	*1	*1/*1 (正常代谢型)	疗效正常
厄贝沙坦、缬沙坦	CYP2C9*3(A>C)	*1	*1/*1 (正常代谢型)	疗效正常
卡托普利、赖诺普利	ACE(I/D)	I	II	疗效增强↑
依那普利、贝那普利、培哚普利、福辛普利	ACE(I/D)	I	II	疗效减弱↓
氢氯噻嗪	ADD1(G>T)	G	GT	疗效正常
呋塞米、螺内酯	ADD1(G>T)	G	GT	疗效减弱↓
美托洛尔、普萘洛尔	ADRB1(G>C)	C	GG	疗效可能减弱↓<br>心动过缓的发生风险增加↑
	CYP2D6(C>T)	C	TT	
比索洛尔、阿替洛尔	ADRB1(G>C)	C	GG	疗效减弱↓

试用水印

三、检测结论

☑常规用药	监测下用药	↓降低剂量用药	↑增加剂量用药	⊗避免用药（换药）
氨氯地平、硝苯地平<br>氯沙坦<br>厄贝沙坦、缬沙坦<br>卡托普利、赖诺普利<br>氢氯噻嗪				依那普利、贝那普利、培哚普利、福辛普利<br>呋塞米、螺内酯<br>美托洛尔、普萘洛尔<br>比索洛尔、阿替洛尔

Supplementary Table S2. Association of individual genetic variants with treatment outcomes in the experimental group (n=1,351)

Gene (risk allele)	Outcome	Carriers (n)	Carriers value	Non-carriers (n)	Non-carriers value	P value	OR (95% CI)
ACE (D)	SBP reduction (mmHg)	753	–26.17 ± 41.43	598	–26.73 ± 13.98	0.726	–
	DBP reduction (mmHg)		–15.44 ± 11.60		–14.81 ± 10.75	0.303	–
	BP control rate (%)		88.18%		85.79%	0.221	1.24 (0.90–1.70)
	Regimen change rate (%)		80.88%		77.42%	0.137	1.23 (0.95–1.61)
*CYP2C9*3 (C)	SBP reduction (mmHg)	128	–25.73 ± 12.99	1223	–26.49 ± 33.68	0.615	–
	DBP reduction (mmHg)		–14.41 ± 12.10		–15.24 ± 11.14	0.463	–
	BP control rate (%)		83.59%		87.49%	0.266	0.73 (0.44–1.20)
	Regimen change rate (%)		75.78%		79.72%	0.351	0.80 (0.52–1.22)
ADRB1 (C)	SBP reduction (mmHg)	1266	–27.14 ± 13.95	85	–15.69 ± 117.07	0.370	–
	DBP reduction (mmHg)		–15.25 ± 11.08		–13.76 ± 13.32	0.317	–
	BP control rate (%)		86.73%		92.94%	0.137	0.50 (0.21–1.16)
	Regimen change rate (%)		79.30%		80.00%	0.988	0.96 (0.55–1.66)
*CYP2D6*10 (T)	SBP reduction (mmHg)	1059	–26.93 ± 13.89	292	–24.58 ± 64.28	0.535	–
	DBP reduction (mmHg)		–14.94 ±		–15.96 ± 10.97	0.164	–

Gene (risk allele)	Outcome	Carriers (n)	Carriers value	Non-carriers (n)	Non-carriers value	P value	OR (95% CI)
			11.30				
	BP control rate (%)		87.16%		86.99%	1.000	1.02 (0.69–1.49)
	Regimen change rate (%)		78.28%		83.22%	0.078	0.73 (0.52–1.02)
*CYP3A5*3 (A)	SBP reduction (mmHg)	689	–27.32 ± 13.80	662	–25.48 ± 43.93	0.302	–
	DBP reduction (mmHg)		–15.71 ± 11.62		–14.58 ± 10.79	0.064	–
	BP control rate (%)		86.79%		87.46%	0.775	0.94 (0.68–1.30)
	Regimen change rate (%)		79.97%		78.70%	0.611	1.08 (0.83–1.41)
CACNA1C (G)	SBP reduction (mmHg)	304	–25.30 ± 13.30	1047	–26.74 ± 35.97	0.283	–
	DBP reduction (mmHg)		–13.56 ± 10.74		–15.62 ± 11.33	**0.004**	–
	BP control rate (%)		88.82%		86.63%	0.365	1.23 (0.82–1.83)
	Regimen change rate (%)		85.20%		77.65%	**0.005**	1.66 (1.17–2.35)
ADD1 (T)	SBP reduction (mmHg)	955	–25.97 ± 37.35	396	–27.49 ± 13.89	0.279	–
	DBP reduction (mmHg)		–15.23 ± 10.83		–14.99 ± 12.16	0.744	–
	BP control rate (%)		85.76%		90.40%	**0.026**	0.64 (0.44–0.94)
	Regimen change rate (%)		80.10%		77.53%	0.321	1.17 (0.88–1.55)

*Values for blood pressure reduction are mean ± standard deviation. P values for continuous

outcomes were calculated by independent samples t-test; P values for categorical outcomes were calculated by chi-square test.

OR (odds ratio) and 95% confidence interval are presented only for categorical outcomes.

Statistically significant results ($P < 0.05$) are shown in bold.

Supplementary Table S3. Baseline characteristics and standardized mean differences between groups

Variable	Experimental group (n=1351)	Control group (n=1351)	Standardized mean difference (SMD)	Balanced (SMD<0.1)
Age (years), mean±SD	59.97±12.73	61.48±11.37	0.125	No
Male, n (%)	780 (57.7%)	756 (56.0%)	0.036	Yes
BMI (kg/m²), mean±SD	24.95±3.32	24.82±3.31	0.038	Yes
Baseline SBP (mmHg), mean±SD	155.45±11.94	156.52±11.46	0.091	Yes
Baseline DBP (mmHg), mean±SD	92.12±11.08	91.75±10.28	0.035	Yes
Diabetes, n (%)	16 (1.2%)	1 (0.1%)	0.140	No
Obesity, n (%)	0	0	0	Yes
Stroke, n (%)	7 (0.5%)	1 (0.1%)	0.082	Yes
Cardiovascular disease, n (%)	22 (1.6%)	14 (1.0%)	0.052	Yes
Dyslipidemia, n (%)	11 (0.8%)	4 (0.3%)	0.070	Yes

SMD = (mean difference)/pooled SD for continuous variables; for binary variables, SMD was calculated using the formula for proportion differences. SMD <0.1 indicates good balance. Age and diabetes showed minor imbalance; all other baseline characteristics were balanced.

Supplementary Table S4. Subgroup analysis by baseline antihypertensive medication status

Subgroup	Outcome	Experimental group	Control group	P value
**Treatment-naïve patients (no baseline medication)**	n=385	n=156		
	SBP reduction (mmHg), mean±SD	-27.48±14.95	-22.03±17.33	0.00068
	DBP reduction (mmHg), mean±SD	-15.36±10.24	-11.04±13.12	0.00029
	BP control rate at week 4	88.05%	39.10%	2.55×10^{-31}
**Previously treated patients (on medication at baseline)**	n=966	n=1195		
	SBP reduction (mmHg), mean±SD	-26.00±37.00	-11.58±13.61	4.44×10^{-29}
	DBP reduction (mmHg), mean±SD	-15.08±11.61	-8.75±11.10	2.61×10^{-36}
	BP control rate at week 4	86.75%	19.75%	5.00×10^{-210}

BP control defined as seated office SBP <140 mmHg and DBP <90 mmHg.

Supplementary Table S5. Treatment intensity and medication pattern changes during the 4-week period

Parameter	Experimental group (n=1351)	Control group (n=1351)	P value
**Treatment adjustments**			
Regimen change rate within 4 weeks	79.35%	32.57%	<0.001
**Combination therapy**			
Baseline combination therapy (≥2 drugs)	20.95%	16.21%	0.0018
Week 4 combination therapy	45.37%	28.72%	4.53×10^{-19}
Change in combination therapy	+24.42%	+12.51%	–
**Monotherapy**			
Baseline monotherapy	79.05%	83.79%	–
Week 4 monotherapy	54.63%	71.28%	–
**Untreated patients**			
Baseline no medication	28.50%	11.55%	5.88×10^{-28}

Drug class utilization

Drug class	Time point	Experimental group	Control group	P value
ARBs	Baseline	25.54%	25.24%	0.895
	Week 4	72.69%	34.49%	7.97×10^{-88}
ACEIs	Baseline	5.11%	4.37%	0.415
	Week 4	4.74%	5.03%	0.789
Beta-blockers	Baseline	5.77%	10.88%	2.19×10^{-6}
	Week 4	14.88%	15.17%	0.872
CCBs	Baseline	55.74%	63.29%	7.55×10^{-5}
	Week 4	53.44%	74.54%	5.16×10^{-30}
Diuretics	Baseline	3.18%	2.52%	0.355
	Week 4	2.59%	3.48%	0.217

P values: McNemar test for within-group changes, chi-square test for between-group comparisons.

Supplementary Table S6. Subgroup analyses of systolic blood pressure reduction and control rate

**Subgroup**	**Stratum**	**Outcome**	**Experimental group**	**Control group**	**P value**
**Age**	<65 years	SBP reduction (mmHg)	-24.85±39.94 (n=818)	-11.61±14.09 (n=828)	1.70×10^{-18}
		BP control rate	84.84%	20.17%	1.74×10^{-151}
	≥65 years	SBP reduction (mmHg)	-28.82±13.64 (n=533)	-14.64±14.88 (n=523)	1.98×10^{-52}
		BP control rate	90.62%	24.86%	2.26×10^{-103}
**Baseline SBP**	<160 mmHg	SBP reduction (mmHg)	-20.03±37.99 (n=858)	-6.77±10.00 (n=811)	5.70×10^{-22}
		BP control rate	90.09%	20.59%	1.96×10^{-179}
	≥160 mmHg	SBP reduction (mmHg)	-37.54±12.33 (n=493)	-21.82±15.43 (n=540)	5.05×10^{-64}
		BP control rate	81.95%	24.07%	1.19×10^{-76}
**Diabetes**	No diabetes	SBP reduction (mmHg)	-26.54±32.44 (n=1335)	-12.79±14.48 (n=1350)	2.91×10^{-43}
		BP control rate	87.42%	22.00%	2.48×10^{-253}
**Obesity**	No obesity	SBP reduction (mmHg)	-26.42±32.29 (n=1351)	-12.78±14.47 (n=1351)	2.52×10^{-43}
		BP control rate	87.12%	21.98%	8.32×10^{-253}

Data are mean ± SD or percentage. P values: t-test for continuous variables, chi-square test for categorical variables.

Supplementary Table S7. Multivariable regression analysis for blood pressure outcomes

Outcome	Model	Covariates	Effect estimate (β for SBP reduction; OR for control)	95% CI	P value
SBP reduction (mmHg)	Linear regression	Age, sex, baseline SBP, baseline DBP	-14.64 (β)	-16.37 to -12.91	9.18×10^{-59}
BP control at week 4 (yes/no)	Logistic regression	Age, sex, baseline SBP, baseline DBP	25.78 (OR)	20.87 to 31.84	9.75×10^{-200}

Reference group: control group. BP control defined as seated office SBP <140 mmHg and DBP <90 mmHg.

Supplementary Table S8. Comparison of effect sizes with previous pharmacogenomics-guided hypertension trials

Study	Study type	Sample size (experimental/control)	Population	Intervention duration	Baseline SBP (mmHg)	BP control rate difference	SBP reduction difference (experimental vs. control)
**Our study**	RCT	1351/1351	Chinese (Hunan), essential hypertension, baseline SBP ~155 mmHg	4 weeks	155	+65.2%	+16.9 mmHg
Xiao et al. (2022) [Ref 13]	RCT	495/165	Chinese (Changsha), baseline SBP ~140 mmHg	4 weeks (ABPM)	137	+4.9%	+5.0 mmHg
Wang et al. (2022)	RCT	~120/120	Chinese, resistant hypertension	12 weeks	~150	+42.0%	+17.0 mmHg
Johnson et al. (2021)	RCT	300/300	European/American, essential hypertension	6 weeks	~145	+12.3%	+6.8 mmHg
Li et al. (2023)	RCT	150/150	Chinese, hypertension with diabetes	4 weeks	~148	+28.5%	+12.5 mmHg

BP control rate difference = (control rate in experimental group) – (control rate in control group). SBP reduction difference = (SBP reduction in experimental group) – (SBP reduction in control group).

Supplementary Table S9 Baseline Balance Between Included and Excluded Patients

Variable	Included Patients (n=2702)	Excluded Patients (n=5989)	Standardized Mean Difference (SMD)	Clinically Meaningful Difference
Age (years)	60.60±12.68	61.50±13.42	0.069	No
SBP (mmHg)	155.99±11.71	155.67±12.43	0.026	No
DBP (mmHg)	91.93±10.69	91.55±11.11	0.035	No
Male Sex (%)	56.85%	54.84%	0.040	No
BMI (kg/m²)	24.95±3.32	24.88±3.41	0.021	No

Supplementary Table S10. Adverse events in the experimental and control groups

Adverse event	Experimental group (n=1351)	Control group (n=1351)	P value*
Dry cough	2 (0.15%)	0	0.4998
Neurotic edema	1 (0.07%)	0	1.0000
Hypotension	0	0	1.0000
Dizziness	1 (0.07%)	0	1.0000
Headache	0	0	1.0000
Hypokalemia	0	0	1.0000
**Any adverse event (≥1)**	**4 (0.3%)**	**0**	**0.1247**

*P values were calculated using Fisher's exact test. No serious adverse events occurred in either group.
